# Genetic and Environmental Risk Factors for Isolated Hemangiomas in Infants

**DOI:** 10.3390/children7100150

**Published:** 2020-09-25

**Authors:** Anna Materna-Kiryluk, Katarzyna Wiśniewska, Barbara Więckowska, Katarzyna Wróblewska-Seniuk, Beata Jaroszewska-Świątek, Ewa Helwich, Anna Latos-Bieleńska

**Affiliations:** 1 Polish Registry of Congenital Malformations, Chair and Department of Medical Genetics, Poznan University of Medical Sciences, 61-701 Poznan, Poland; alatos@ump.edu.pl; 2 Department of Preventive Medicine, Epidemiology Unit, Poznan University of Medical Sciences, 61-701 Poznan, Poland; kwisniewska@ump.edu.pl; 3 Department of Computer Science and Statistics, Poznan University of Medical Sciences, 61-701 Poznan, Poland; basia@ump.edu.pl; 4 Department of Newborns’ Infectious Diseases, Poznan University of Medical Sciences, 61-701 Poznan, Poland; kwroblewska@ump.edu.pl; 5 Department of Clinical Pediatrics, University of Warmia and Mazury in Olsztyn, Children’s Hospital in Olsztyn, 10-651 Olsztyn, Poland; swiatekfam@poczta.onet.pl; 6 Deparment of Neonatology and Neonatal Intensive Care, Institute of Mother and Child, 01-211 Warszawa, Poland; ewa.helwich@imid.med.pl

**Keywords:** infant hemangiomas, risk factors, hereditary predisposition, vascular malformations

## Abstract

The goal of this analysis is to identify risk factors for infantile hemangiomas (IH) to better delineate hemangioma predisposition. We analyzed live birth children with isolated cutaneous hemangioma that were reported to the Polish Registry of Congenital Malformations from across Poland between the years 1998 and 2016. Lower birthweight and gestational age were the most significant risk factors associated with IH. We also observed a trend for a higher risk of IH with a lower level of maternal and paternal education. Moreover, mothers with IH have a higher probability of having a child with IH compared to fathers. However, this association is only present when the child is female. Similarly, a higher risk of hemangioma in a female child is found among mothers having relatives of the first degree with IH, compared to fathers with a similar pedigree. Our results suggest the role of exogenous factors in the etiology of IH. The analysis of familial cases suggests a multifactorial model of inheritance. The study indicates that female gender is an important risk factor for the expression of familial IH. Potential interaction of genetic risk factors with exposure to female sex hormones may play a role in the development of IH.

## 1. Introduction

The most frequent tumors occurring in infants are hemangiomas. Some of them, due to their localization, can pose a serious threat to the child’s health. Most commonly, however, hemangiomas create a problem of a cosmetic nature. Frequently, their occurrence is sporadic, but in some cases, they are familial. In recent years, an increase in the frequency of hemangiomas in the world has been observed [1,2]. The pathogenesis and precise etiology of infantile hemangiomas (IH) are not entirely known and therefore, the identification of risk factors can be useful. Many risk factors have been identified, including maternal vaginal bleeding during the first trimester of pregnancy, progesterone use, preeclampsia, and placenta previa [3,4]. Some studies have also suggested that IH may be associated with the use of in vitro fertilization [4,5]. Moreover, neonatal phototherapy has also been described to increase the risk of IH, but further confirmation of these findings is needed [6]. Another study demonstrated that infantile vascular anomalies express follicle-stimulating hormone receptor on their endothelium, suggesting that follicle stimulating hormone (FSH) may be involved in the pathogenesis of IH, especially given that the secretion of FSH correlates with the growth pattern of vascular malformations [7]. The risk factors for IH in the Polish population have not been studied previously. The goal of this paper is to comprehensively characterize the impact of parental demographic factors and predisposition to hereditary IH.

## 2. Materials and Methods

The subjects of analysis were live birth children with isolated cutaneous hemangioma born in Poland and registered in the country-wide Polish Registry of Congenital Malformations (PRCM) between 1998 and 2016. Standardized questionnaires reporting congenital malformations were used as the primary source of information. The questionnaires were completed by physicians from neonatal, pediatric and obstetric wards, and the information was collected and entered into the centralized Registry Database. The organizational structure of PRCM, methods of data collection and verification have been published previously [8]. IH coding was done according to the ICD-10 coding scheme. Our analysis excluded children with known syndromes and children with IH accompanied by other birth defects. In total, our analysis included 4538 children with isolated IH. Within this group, 517 cases of IH occurred in families (relatives of the first, second, and further degrees). In the remaining 4021 children, there was no family history of IH, and this group is referred to as sporadic isolated cases of IH.

The control group consisted of healthy children born in the same time period as the analyzed group. The primary source of information was from parallel standardized questionnaires completed by midwives. Altogether, 19,160 mother-child dyads volunteered to participate in the registry for the purpose of serving as controls. Because the characteristics of the volunteering mothers from the control group did not fully reflect the Polish population in terms of age, education and area of residence, we randomly sub-selected controls from this group that are more representative of the general Polish population as described by Polish Statistical Office (GUS). In order to select mothers from the recruited control group, the recruited group was divided according to mother’s age, level of education, and urban/rural strata. Random sampling was then conducted within each stratum using the proportionate allocation method, such that the relative size of each stratum was matched to the population data. Briefly, the target size for each stratum was first determined, based on the relative population distribution across the strata and the target control group size (equal to the case group size). This was followed by a random control selection into each stratum from a pool of available controls, which was achieved by a random number generation for each control, followed by sorting and sequential selection of the top-ranking controls to each stratum up to the target stratum size. As a result, the final sample fraction for each stratum was proportional to this fraction in the general population. This method was used to sub-select the 4538 controls used in this study.

Analysis of the influence of individual risk factors on the frequency of IH occurrence was conducted using logistic regression. In the first stage, a univariable model was created. In the second stage, the regression model was extended to multiple variables reflective of additional factors that showed significant connection with IH occurrence in univariate analysis. In order to avoid collinearity, no simultaneous correction was done with regards to strongly inter-correlated maternal and paternal factors, such as age or level of education. Accordingly, the models were limited solely to maternal-related factors. The variables of birthweight (BW) and fetus’ age (GA) were categorized as follows: GA ≤ 36 and BW ≤ 2499, GA ≤ 36 and BW ≥ 2499, GA ≥ 36 and BW ≤ 2499, GA ≥ 36 and BW ≥ 2499. We considered *p*-values of less than 0.05 as statistically significant. All analyses were conducted in the PQStat v1.6.6 statistical program.

## 3. Results

The most common site of IH in infants was head and neck (34.4%), extremities (31.2%) and trunk (16.7%) (Table 1). Singular lesions were present in 73.6% of affected infants, while multiple IH (2–5 lesions) occurred in 23.6% of cases. Only 2.8% infants had more than five lesions. 

The comparison of characteristics of children with isolated IH and healthy children from the control group is presented in Table 2. The results show that IH was more common among females OR (odds ratio) 1.43, 95% CI (confidence interval) 1.31, 1.57), regardless of birthweight, week of pregnancy and maternal age or education.

Lower birthweight and gestational age were the most significant risk factors associated with IH. Prematurity (≤36 months) and low birthweight (≤2499 g) were significantly more common in children with IH compared to healthy population controls (OR 1.75, 95% CI 1.37, 2.25). Additional independent factors significantly associated with an increased risk of having a child with IH included younger maternal age (OR 1.13, 95% CI 1.02, 1.26) and high gravidity of equal or over 2 (OR 1.11, 95% CI 1.0, 1.23). We also observed a trend for a higher risk of IH with a lower level of maternal and paternal education. For primary and basic occupational education, the risk of having a child with IH was almost twice as high as for parents with higher education (mother: OR 1.81, 95% CI 1.61, 2.04; father: OR 1.81, 95% CI 1.55, 2.12), and for high school education over 1.5 times higher (mother: OR 1.59, 95% CI 1.43, 1.78; father: OR 1.56, 95% CI 1.36, 1.79). There was no correlation between urban/rural residence, twin pregnancy, the number of previous miscarriages, terminations of pregnancy, stillbirths or maternal tobacco use.

In our study, approximately 89% of IH cases occurred sporadically, and 11% of cases were familial. The comparison of demographic and maternal factors in familial and sporadic groups demonstrates that the risk of having a child with IH in a sporadic group (compared to the familial group) is significantly higher for less educated mothers with primary and basic occupational education: OR 1.34, 95% CI 1.04, 1.71, and for high school education: OR 1.27, 95% CI 1.0, 1.61 (Table 3). 

The risk of having a child with IH in the familial group (compared to sporadic) was about 1.7 times higher when it is ≥2 pregnancy (OR 0.58, 95% CI 0.47, 0.72). In the univariate analysis, the risk of having a child with IH in the familial group (compared to sporadic) was about 1.3 times higher for older fathers (OR 0.79, 95% CI 0.63, 0.98). However, no statistical significance in the fully adjusted model was found. The risk of having a child with IH in the familial group was more than twice as high compared to the sporadic group when it was a twin pregnancy (OR 0.47, 95% CI 0.28, 0.80), but no statistical significance was found in the adjusted model.

Across all categories, female sex represented an important independent risk factor for IH across all categories, but also among familial cases. In 358 cases out of 517 in children from familial IH, at least one first degree relative had IH (Table 4). 

There were 207 of 358 (57.8%) affected females in the familial disease group with an affected first-degree relative. Notably, among familial cases, mothers of children with IH were affected significantly more often compared to fathers (frequency of affected mothers = 73/358 (20.4%), frequency of affected fathers = 40/358 (11.2%), DIFF (difference in proportions) 9.2%, 95% CI 3.9%, 14.5%). Moreover, for all reported familial cases, IH were transmitted more commonly from the maternal rather than paternal side (maternal transmission frequency = 99/517 (19.1%), paternal transmission frequency = 58/517 (11.2%), DIFF 7.9%, 95% CI 3.6%, 12.3%).

This relationship became even more striking in the analysis of female cases with familial disease. In these cases, the mothers were affected significantly more often compared to the fathers (frequency of affected mothers = 38/207 (18.4%), frequency of affected fathers = 17/207 (8.2%), DIFF 10.1%, 95% CI 3.6%, 16.7%), and the transmission of IH was considerably more frequent from the maternal side (maternal transmission frequency = 54/207 (26.1%), paternal transmission frequency = 23/207 (11.1%), DIFF 15.0%, 95% CI 7.5%, 22.3%). In the analysis of male cases, no such relationship was observed.

## 4. Discussion

The etiopathogenesis of IH remains largely unexplained and the relative contributions of environmental and genetic factors in the etiology of IH is not well understood. This paper describes the risk factors for sporadic and familial forms of isolated IH, highlighting strong effects of female sex on the risk, and providing support for a maternal transmission of this trait.

The PRCM collects data on congenital malformations diagnosed among Polish children less than 2 years of age. In this study, we presented the prenatal characteristics of a large group of patients with isolated IH. Our study is one of the largest case-control studies examining fetal risk factors (birthweight, gestation age, sex), parental factors (age, education, urban/rural residence, previous miscarriages, terminations of pregnancy and stillbirths and maternal tobacco use) and assessing the relationship of people with IH. The strength of this study is the size of the examined population, which is the largest described until now in the literature. The large numbers of IH reported to PRCM enabled us to carry out comparison between familial and sporadic (non-familial) forms of IH. In addition, we included a well-characterized group of healthy control births to further validate risk factors specific to IH. Our data were reviewed by clinical geneticists.

Nevertheless, several limitations of our study need to be discussed. First, our analysis was limited to the records of infants in PRCM. Unfortunately, IH cases are not included in the mandatory reporting to either PRCM or other European registries within EUROCAT. Thus, we were not able to evaluate the overall incidence of IH in the Polish population. Another limitation was the incompleteness of some of our data. The information on mothers’ illnesses, complications during pregnancy, maternal alcohol and drug abuse in our database is incomplete. Although these potential risk factors have been suggested by others [9], they were not analyzed in our study.

As many as 89% of all reported IH occurred sporadically and the results of our analysis strongly suggest the role of exogenous factors in the etiology of this trait. Similar to previous reports [10,11,12], our results demonstrate that isolated IH are often associated with prematurity and low birthweight. The risk of developing IH in children born preterm with low birthweight suggests the participation of common factors in the etiology of prematurity and IH. Some authors suggested that placental complications in utero may have an impact on both prematurity and hemangioma development [13]. Placental anomalies were noted more often in mothers of infants with IH compared with pregnancies with unaffected infants [3,12]. Some authors suggested that IH may be derived from placental tissue [14]. Placental insufficiency may cause hypoxia, and this, in turn, may represent a potential risk factor of hemangioma development [15,16].

More recently, genome-wide association studies (GWAS) in affected IH children and their families identified single nucleotide polymorphisms related to Nitric Oxide Synthase Trafficking (NOSTRIN), a trafficking inducer protein involved in the eNOS/hypoxia pathway [17]. Therefore, the hypothesis has been put forward about the relationship between hypoxia and the development of IH [12,16]. Our results supporting the association of prematurity with IH may be relevant to this hypothesis.

We also observed a trend for a higher risk of IH with a lower level of maternal and paternal education. Parents with lower levels of education have a higher risk of having a child with IH. The less educated parents may be more likely to be exposed to harmful influences of the external environment through less awareness of environmental threats and their avoidance. This may include, for example, living in more polluted areas, having jobs more vulnerable to harmful factors, and less pro-health lifestyle. In univariate analyses of sporadic cases, young maternal and paternal ages represented additional risk factors for IH. This result however, was confirmed in the adjusted model, only with regard to young maternal age. We note that these results are not consistent with some reports indicating an advanced age of the mother as a risk factor for having a child with IH [3,18].

A younger maternal age may be associated with specific health behaviors, such as unplanned pregnancies, or alcohol and drug consumption. The identification of risk factors for IH by the investigation of age-specific behavioral factors may lead to improved preventive strategies, and may help to establish the disease etiology and pathophysiology.

One of the most striking findings of our study was a strong female predominance among the cases, with a female-to-male sex ratio of 1.43. Sex dimorphism in the determination of specific defects can provide additional insights into disease pathogenesis [19]. A significant risk factor of IH in females has previously been reported by other authors [3,10,12,20]. Our study also demonstrates that among familial cases of isolated IH, the trait is most likely inherited from a maternal side of the family. However, this observation is only true if the affected child is a female. Presently, the reasons for female predominance in IH and for the observed maternal line of transmission are unclear. No predisposing genetic mutations or variants were found on sex chromosomes. However, several studies have shown that estrogen can contribute to hemangioma proliferation [21,22]. A study by Zhang et al. provides information about the potential mechanisms of E2 action on IHs [21]. Another study by Maclellan et al. suggested that FSH may be an important sex-specific factor, because FSH receptors are strongly expressed by the endothelium of infantile vascular anomalies, and the secretion of FSH correlates with the growth pattern of vascular malformations [7]. Thus, current data strongly suggest the potential role of female sex hormones in the pathogenesis of IH, but the precise molecular determinants of the observed sexual dimorphism will require further mechanistic studies.

## 5. Conclusions

Our work points to the important contribution of factors related to female sex, and their potential interaction with inherited risk. The hormonal contribution to the development of IH represents one possible explanation of these findings.

## Figures and Tables

**Table 1:** Localization of infantile hemangiomas (IH).

Localization of IH	*n* = 4538
	Count (%)
Head and neck	1561 (34.4%)
Limbs	1414 (31.2%)
Trunk	759 (16.7%)
Head and neck + Trunk	113 (2.5%)
Head and neck + Limbs	66 (1.5%)
Head and neck + Trunk + Limbs	59 (1.3%)
Trunk + Limbs	298 (6.6%)
Unknown	268 (5.9%)

**Table 2:** Characteristics of children and their mothers with IH and in control group.

Characteristic:	Controls	Isolated IH				
	*n* = 4538	*n* = 4538	Unadjusted Model		Adjusted Model (GA and BW. Sex. Maternal Age and Maternal Education)	
	Count (%)	Count (%)	*p*-Value	OR Crude (95%CI)	*p*-Value	OR Adjusted (95%CI)
**Gestational age (GA) in weeks and**						
**Birthweight (BW) in grams**						
GA ≤ 36 and BW ≤ 2499	111 (2.4%)	217 (4.8%)	<0.0001	2.01 [1.59, 2.54]	<0.0001	1.75 [1.37, 2.25]
GA ≤ 36 and BW ≥ 2499	301 (6.6%)	284 (6.3%)	0.7304	0.97 [0.82, 1.15]	0.2066	0.89 [0.74, 1.07]
GA ≥ 36 and BW ≤ 2499	83 (1.8%)	108 (2.4%)	0.0481	1.34 [1.00, 1.79]	0.7403	1.06 [0.77, 1.45]
GA ≥ 36 and BW ≥ 2499	4043 (89.1%)	3929 (86.6%)	reference		reference	
**Sex**						
Male	2342 (51.6%)	1910 (42.1%)	reference		reference	
Female	2186 (48.2%)	2599 (57.3%)	<0.0001	1.46 [1.34, 1.58]	<0.0001	1.43 [1.31, 1.57]
**Place of residence** ^§^						
Rural	1888 (41.6%)	1917 (42.2%)	0.1711	1.06 [0.98, 1.15]	0.1592	0.94 [0.85, 1.03]
Urban	2650 (58.4%)	2538 (55.9%)	reference		reference	
**Maternal age in years** ^§^						
≤24	1123 (24.7%)	1378 (30.4%)	<0.0001	1.37 [1.24, 1.50]	0.0209	1.13 [1.02, 1.26]
25–34	2863 (63.1%)	2569 (56.6%)	reference			
≥35	552 (12.2%)	482 (10.6%)	0.6886	0.97 [0.85, 1.11]	0.3867	0.94 [0.81, 1.08]
**Paternal age in years** ^§^						
≤24	543 (12.0%)	645 (14.2%)	<0.0001	1.31 [1.15, 1.48]	0.8462	1.02 [0.87, 1.18]
25–34	2875 (63.4%)	2614 (57.6%)	reference		reference	
≥35	1032 (22.7%)	891 (19.6%)	0.3301	0.95 [0.86, 1.05]	0.5533	0.96 [0.84, 1.09]
**Maternal education** ^§^						
Primary and basic occupational	1140 (25.1%)	1222 (26.9%)	<0.0001	1.93 [1.72, 2.15]	<0.0001	1.81 [1.61, 2.04]
High school	1597 (35.2%)	1481 (32.6%)	<0.0001	1.67 [1.50, 1.85]	<0.0001	1.59 [1.43, 1.78]
Higher	1801 (39.7%)	1002 (22.1%)	reference		reference	
**Paternal education** ^§^						
Primary and basic occupational	1526 (33.6%)	1597 (35.2%)	<0.0001	2.26 [2.02, 2.54]	<0.0001	1.81 [1.55, 2.12]
High school	1425 (31.4%)	1202 (26.5%)	<0.0001	1.82 [1.62, 2.06]	<0.0001	1.56 [1.36, 1.79]
Higher	1438 (31.7%)	665 (14.7%)	reference		reference	
**Gravidity** ^§^						
1	2001 (44.1%)	2027 (44.7%)	reference		reference	
≥2	2337 (51.5%)	2407 (53.0%)	0.6984	1.02 [0.93, 1.11]	0.0408	1.11 [1,0, 1.23]
**Twin pregnancy** ^§^						
No	4460 (98.3%)	4453 (98.1%)		reference		reference
Yes	73 (1.6%)	85 (1.9%)	0.3395	0.86 [0.63, 1,18]	0,5528	0.90 [0.64, 1.27]
**Previous miscarriages** ^‡ §^						
No	1746 (74.7%)	1829 (76.0%)	reference		reference	
Yes	531 (22.7%)	512 (21.3%)	0.2391	0.92 [0.80, 1.06]	0.6641	0.97 [0.83, 1.12]
**Previous stillbirths** ^‡§^						
No	2190 (93.7%)	2215 (92%)	reference		reference	
Yes	33 (1.4%)	36 (1.5%)	0.7554	1.08 [0.67, 1.74]	0.5039	1.18 [0.72, 1.94]
**Maternal tobacco use** ^§^						
No	3846 (84.8%)	2446 (53.9%)	reference		reference	
Yes	254 (5.6%)	175 (3.9%)	0.4308	1.08 [0.89, 1.32]	0.2254	0.88 [0.71, 1.08]

^‡^ 2nd pregnancy and subsequent. ^§^ marginal totals are different because of missing values. Odds ratio (OR), confidence interval (CI), birthweight (BW), fetus’ age (GA), infantile hemangiomas (IH).

**Table 3:** Characteristics of children and their mothers with familial and non-familial IH.

Characteristic:	Familial	Non-Familial	Non-Familial vs. Familial			
	*n* = 517	*n* = 4021	Unadjusted Model		Adjusted Model (Adjusted for Paternal Age. Maternal Education and Gravidity)	
	Count (%)	Count (%)	*p*-Value	OR Crude (95% CI)	*p*-Value	OR Adjusted (95%CI)
**Gestational age (GA) in weeks and**						
**Birthweight (BW) in grams**						
GA ≤ 36 and BW ≤ 2499	26 (5.0%)	191 (4.8%)	0.7941	0.95 [0.62, 1.44]	0.9696	0.99 [0.61, 1.61]
GA ≤ 36 and BW ≥ 2499	31 (6.0%)	253 (6.3%)	0.8029	1.05 [0.71, 1.54]	0.9050	0.98 [0.64, 1.47]
GA ≥ 36 and BW ≤ 2499	12 (2.3%)	96 (2.4%)	0.9251	1.03 [0.56, 1.89]	0.6335	0.85 [0.44, 1.64]
GA ≥ 36 and BW ≥ 2499	448 (86.7%)	3481 (86.6%)	reference		reference	
**Sex** ^§^						
Male	211 (40.8%)	1699 (42.3%)	reference		reference	
Female	306 (59.2%)	2293 (57.0%)	0.4493	0.93 [0.77, 1.12]	0.6269	0.95 [0.78, 1.16]
**Place of residence** ^§^						
Rural	211 (40.8%)	1706 (42.4%)	0.3557	1.09 [0.91, 1.32]	0.3143	1.11 [0.91, 1.36]
Urban	302 (58.4%)	2236 (55.6%)	reference		reference	
**Maternal age in years**						
≤24	141 (27.3%)	1237 (30.8%)	0.1298	1.18 [0.95, 1.45]	0.6907	0.95 [0.75, 1.21]
25-34	304 (58.8%)	2265 (56.3%)	reference		reference	
≥35	67 (13.0%)	415 (10.3%)	0.2031	0.83 [0.63, 1.1]	0.7697	0.96 [0.71, 1.29]
**Paternal age in years** ^§^						
≤24	77 (14.9%)	568 (14.1%)	0.7848	0.96 [0.74, 1.26]	0.1711	0.81 [0.61, 1.09]
25-34	302 (58.4%)	2312 (57.5%)	reference		reference	
≥35	127 (24.6%)	764 (19.0%)	0.0340	0.79 [0.63, 0.98]	0.5169	0.92 [0.73, 1.17]
**Maternal education** ^§^						
Primary and basic occupational	154 (29.8%)	1068 (26.6%)	0.0327	1.30 [1.02, 1.65]	0.0233	1.34 [1.04, 1.71]
High school	184 (35.6%)	1297 (32.3%)	0.0179	1.32 [1.05, 1.66]	0.0463	1.27 [1.00, 1.61]
Higher	158 (30.6%)	844 (21.0%)	reference		reference	
**Paternal education** ^§^						
Primary and basic occupational	216 (41.8%)	1381 (34.3%)	0.1347	1.21 [0.94, 1.56]	0.5548	1.07 [0.86, 1.34]
High school	162 (31.3%)	1040 (25.9%)	0.1466	1.22 [0.93, 1.59]	0.2578	0.85 [0.65, 1.12]
Higher	106 (20.5%)	559 (13.9%)	reference		reference	
**Gravidity** ^§^						
1	181 (35.0%)	1846 (45.9%)	reference		reference	
≥2	329 (63.6%)	2078 (51.7%)	<0.0001	0.62 [0.51, 0.75]	<0.0001	0.58 [0.47, 0.72]
**Twin pregnancy** ^§^						
No	3954 (98.3%)	499 (96.5%)	reference		reference	
Yes	67 (1.7%)	18 (3.5%)	0.0051	0.47 [0.28, 0.80]	0.2963	0.72 [0.39, 1.33]
**Previous miscarriages** ^‡ §^						
No	243 (73.9%)	1586 (76.3%)	reference		reference	
Yes	80 (24.3%)	432 (20.8%)	0.1754	0.83 [0.63, 1.09]	0.5883	0.92 [0.69, 1.23]
**Previous stillbirths** ^‡§^						
No	317 (96.4%)	1898 (91.3%)	reference		reference	
Yes	5 (1.5%)	31 (1.5%)	0.9427	1.04 [0.4, 2.68]	0.4888	1.45 [0.51, 4.16]
**Maternal tobacco use** ^§^						
No	348 (67.3%)	2098 (52.2%)	reference		reference	
Yes	32 (6.2%)	143 (3.6%)	0.1421	0.74 [0.5, 1.11]	0.0567	0.66 [0.43, 1.01]

^‡^ 2nd pregnancy and subsequent. ^§^ marginal totals are different because of missing values. NA—not applicable.

**Table 4:** Number and percentage of children who had IH in the pedigree.

Relatives with IH	Sex of the Child			
	Male		Female	
	*n* = 151	(100%)	*n* = 207	(100%)
Mother N_M_ = 73	35	(23.2%)	38	(18.4%)
Father N_F_ = 40	23	(15.2%)	17	(8.2%)
N_M_/N_F_ = 1.83	1.52		2.24	
*p* = 0.0025 DIFF[95%CI] = 9.2% [3.9%, 14.5%]	*p* = 0.1480 DIFF[95% CI] = 7.9% [−1.0%, 16.8%]		*p* = 0.0065 DIFF[95% CI] = 10.1% [3.6%, 16.7%]	
Pedigree of mother *n* = 99	45	(29.8%)	54	(26.1%)
Pedigree of father *n* = 58	35	(23.2%)	23	(11.1%)
N_M_/N_F_ = 1.71	1.29		2.35	
*p* = 0.0013 DIFF[95%CI] = 7.9% [3.6%, 12.3%]	*p* = 0.3143 DIFF[95%CI] = 6.6% [−3.3%, 16.4%]		*p* = 0.0006 DIFF[95%CI] = 15.0% [7.5%, 22.3%]	

DIFF[95%CI]—difference in proportions with 95% confidence interval. N_M_ count for the male sex; N_F_ count for the female sex; DIFF: difference in proportions_._

## References

[1] Seiffert A., Schneider M., Roessler J., Larisch K., Pfeiffer D. (2017). Incidence, Treatment Patterns, and Health Care Costs of Infantile Hemangioma: Results of a Retrospective German Database Analysis. Pediatric Dermatol..

[2] Anderson K.R., Schoch J.J., Lohse C.M., Hand J.L., Davis D.M., Tollefson M.M. (2016). Increasing incidence of infantile hemangiomas (IH) over the past 35 years: Correlation with decreasing gestational age at birth and birth weight. J. Am. Acad. Dermatol..

[3] Haggstrom A.N., Drolet B.A., Baselga E., Chamlin S.L., Garzon M.C., Kimberly A., Horii A.W., Lucky A.J., Mancini D.W.M., Hemangioma Investigator Group (2007). Prospective study of infantile hemangiomas: Demographic, prenatal, and perinatal characteristics. J. Pediatrics.

[4] Chen X.D., Ma G., Chen H., Ye X.X., Jin Y.B., Lin X.X. (2013). Maternal and perinatal risk factors for infantile hemangioma: A case-control study. Pediatric Dermatol..

[5] Dickison P., Christou E., Wargon O. (2011). A prospective study of infantile hemangiomas with a focus on incidence and risk factors. Pediatric Dermatol..

[6] Auger N., Ayoub A., Lo E., Luu T.M. (2019). Increased risk of hemangioma after exposure to neonatal phototherapy in infants with predisposing risk factors. Acta Paediatr..

[7] Maclellan R., Vivero M., Purcell P., Kozakewich H., DiVasta A., Mulliken J., Fishman S., Greene A. (2014). Expression of follicle-stimulating hormone receptor in vascular anomalies. Plast. Reconstr. Surg..

[8] Latos-Bieleńska A., Materna-Kiryluk A. (2005). Polish Registry of Congenital Malformations—Aims and organization of the registry monitoring 300,000 births a year. J. Appl. Genet..

[9] Kim E.J., Park H.S., Yoon H.S., Cho S. (2015). Maternal and Perinatal Factors of Importance for Occurrence and Severity of Infantile Haemangioma. Acta Derm. Venereol..

[10] Drolet B.A., Esterly N.B., Frieden I.J. (1999). Hemangiomas in children. N. Engl. J. Med..

[11] Drolet B.A., Swanson E.A., Frieden I.J., Hemangioma Investigator Group (2008). Infantile hemangiomas: An emerging health issue linked to an increased rate of low birth weight infants. J. Pediatrics.

[12] Munden A., Butschek R., Tom W.L., Marshall J.S., Poeltler D.M., Krohne S.E., Krohne A.B., Alió M., Ritter D.F., Friedlander V. (2014). Prospective study of infantile haemangiomas: Incidence, clinical characteristics and association with placental anomalies. Br. J. Dermatol..

[13] Boye E., Jinnin M., Olsen B.R. (2009). Infantile hemangioma: Challenges, new insights, and therapeutic promise. J. Craniofacial Surg..

[14] Ritter M.R., Butschek R.A., Friedlander M., Friedlander S.F. (2007). Pathogenesis of infantile haemangioma: New molecular and cellular insights. Expert Rev. Mol. Med..

[15] Colonna V., Resta L., Napoli A., Bonifazi E. (2010). Placental hypoxia and neonatal haemangioma: Clinical and histological observations. Br. J. Dermatol..

[16] Drolet B.A., Frieden I.J. (2010). Characteristics of infantile hemangiomas as clues to pathogenesis: Does hypoxia connect the dots?. Arch. Dermatol..

[17] Kwon E.M., Siegel D.H., Broeckel U., North P.E., Worthey E., Chiu Y.E., Drolet B.A. (2012). Functional analysis of candidate genes identified by genomewide association study (GWAS) of infantile hemangiomas (IH). J. Investig. Dermatol..

[18] Schoch J.J., Hunjan M.K., Anderson K.R., Lohse C.M., Hand J.L., Davis D.M.R., Megha M. (2018). Tollefson. Temporal trends in prenatal risk factors for the development of infantile hemangiomas. Pediatric Dermatol..

[19] Michalski A.M., Richardson S.D., Browne M.L., Carmichael S.L., Canfield M.A., VanZutphen A.R., Anderka M.T., Marshall E.G., Druschel C.M. (2015). Sex ratios among infants with birth defects, National Birth Defects Prevention Study, 1997–2009. Am. J. Med. Genet. Part A..

[20] Haggstrom A.N., Drolet B.A., Baselga E., Chamlin S.L., Garzon M.C., Horii K.A., Anne W.L., Anthony J.M., Denise W.M., Brandon N. (2006). Prospective study of infantile hemangiomas: Clinical characteristics predicting complications and treatment. Pediatrics.

[21] Zhang L., Wu H.W., Yuan W., Zheng J.W. (2017). Estrogen-mediated hemangioma-derived stem cells through estrogen receptor-α for infantile hemangioma. Cancer Manag. Res..

[22] Sun Z.Y., Yang L., Yi C.G., Zhao H., Han D.L., Yang T., Wang L., Nie C.L., Zhang G.Y., Yin G.Q. (2008). Possibilities and potential roles of estrogen in the pathogenesis of proliferation hemangiomas formation. Med. Hypotheses.

